# A genetic screen in *Drosophila* reveals the role of fucosylation in host susceptibility to *Candida* infection

**DOI:** 10.1242/dmm.049218

**Published:** 2022-05-09

**Authors:** Marcus T. Glittenberg, Ilias Kounatidis, Magda Atilano, Petros Ligoxygakis

**Affiliations:** 1 Department of Biochemistry, University of Oxford, South Parks Rd, Oxford OX1 3QU, UK

**Keywords:** Candida albicans, *Drosophila*, Genetic screen, Fucosylation, Host-pathogen interaction

## Abstract

*Candida* infections constitute a blind spot in global public health as very few new anti-fungal drugs are being developed. Genetic surveys of host susceptibilities to such infections using mammalian models have certain disadvantages in that obtaining results is time-consuming, owing to relatively long lifespans, and these results have low statistical resolution because sample sizes are usually small. Here, we report a targeted genetic screening of 5698 RNAi lines encompassing 4135 *Drosophila* genes with human homologues, several of which we identify as important for host survival after *Candida albicans* infection. These include genes in a variety of functional classes encompassing gene expression, intracellular signalling, metabolism and enzymatic regulation. Analysis of one of the screen hits, the infection-induced α-(1,3)-fucosylase FucTA, showed that N-glycan fucosylation has several targets among proteins involved in host defence, which provides multiple avenues of investigation for the mechanistic analysis of host survival to systemic *C. albicans* infection.

## Abstract (precis)

**Summary:** Genes implicated in host survival to *Candida albicans* infection reveal the role of fucosylation in the turnover and/or stability of proteins required for host defence after immune challenge.

## INTRODUCTION

*Candida albicans* (*C. albicans*) is the fourth most common cause of bloodstream infections in developed countries. Invasive *C. albicans* infection causes 5.4 times more deaths than MRSA in the UK and is a major cause of hospital-associated morbidity (UK Health Protection Agency, 2018). The estimated excess medical costs attributed solely to nosocomial candidemia in the USA approaches $1.4 billion per year (Benedict et al., 2019). Therapeutic options are limited and becoming less effective due to the spread of drug-resistant strains. In addition, attempts to create effective fungal vaccines have failed. Therefore, new strategies are needed to stimulate host immunity against *C. albicans*. This requires us to understand the mechanisms of host-pathogen interaction beyond immune recognition receptors and the cells involved, and to define how immunity to infection integrates with host physiology and impacts survival.

Central to the host defence against *C. albicans* infection in humans is innate immunity (reviewed by Salazar and Brown, 2018). Toll-like receptors (TLRs) and the identification of dectin 1 as a β-glucan receptor paved the way for the discovery of new receptors involved in fungal recognition, their downstream signalling pathways and their subsequent cellular responses. However, we still lack a holistic view of host survival following *C. albicans* infection at the whole-organism level (Lionakis and Levitz, 2018). In this context, a more-accessible genetically tractable host model, such as *Drosophila,* could offer significant insights into the process of host survival following systemic infection.

The main tenant in this argument is the evolutionary conservation between *Drosophila* and mammalian immunity centred on Toll, TLRs and NF-κB signalling. This is an ancient signalling mechanism with significant traces in choanoflagellates (e.g. Woznica et al., 2021) and therefore probably in the last common ancestor of eukaryotes (reviewed by Richter and Levin, 2019). With these receptors, the innate immune system senses the invasion of pathogenic microorganisms. Unlike its mammalian counterparts, *Drosophila* Toll is activated by an endogenous cytokine-like ligand, the Nerve Growth Factor homologue Spz (Weber et al., 2003). Spz is processed to its active form by the Spz-Processing Enzyme (SPE) (Jang et al., 2006). Two serine protease cascades converge on SPE: one triggered by bacterial or fungal serine proteases through the host serine protease Persephone (Ligoxygakis et al., 2002; Gottar et al., 2006; Issa et al., 2018); and a second activated by host receptors that recognize bacterial or fungal cell wall through bacterial peptidoglycan or β-glucan recognition, respectively (Gottar et al., 2006; El Chammy et al., 2008).

When the recognition signal reaches the cell surface, it is communicated intracellularly via the Toll receptor and a membrane-bound receptor-adaptor complex, including Myd88, Tube (as an IRAK4 functional equivalent) and the Pelle kinase (as an IRAK1 functional homologue) (Marek and Kagan, 2012; Daigneault et al., 2013). Transduction of the signal culminates in the phosphorylation of the IκB homologue Cactus. This modification requires the fly βTrCP protein Slimb and targets Cactus for degradation (Daigneault et al., 2013), leaving the NF-κB homologue DIF to move to the nucleus and regulate hundreds of target genes, including a battery of powerful antimicrobial peptides (AMPs) (Rutschmann et al., 2000). Recent work has shown that loss of two of these Toll pathway-controlled AMP genes, *metchnikowin* and *drosomycin* renders flies susceptible to *C. albicans* (Hanson et al., 2019). This underlines the specificity of these effector molecules towards this opportunistic fungus and hints towards a more ancient host-pathogen relationship than hitherto suspected. Although loss of Myd88 does not render humans susceptible to fungal infection (von Bernuth et al., 2008), studies have suggested that different human TLRs are able to activate specific arms of the antifungal defence, mainly in collaboration with dectin 1, while polymorphisms in several TLRs, including TLR1, TLR2, TLR3, TLR4, TLR6 and TLR9, have been associated with increased risk of fungal infections in immunocompromised individuals (reviewed by Cunha et al., 2010). This suggests that TLRs are not primarily required for antifungal immunity in humans, but that under conditions of altered immunity their role becomes more apparent.

In *Drosophila*, an intact Toll pathway is important for clearing *C. albicans* systemic infection (Glittenberg et al., 2011). Moreover, independently infecting flies and mice with a series of clinical *C. albicans* isolates, generates the same virulence ranking in both hosts when using host survival time as a metric (Glittenberg et al., 2011). This finding clearly demonstrates that *Drosophila* can be used as a valid alternative host model to evaluate *C. albicans* virulence *in vivo* and help define novel restriction factors of infection at the level of the whole organism.

To this end, we have conducted a large tissue-specific genetic screen in *Drosophila* to identify host genes that, when silenced (through RNAi), compromise or enhance survival to *C. albicans* infection. In total, we screened 5698 RNAi lines encompassing 4135 *Drosophila* genes with human homologues. These human homologues were selected: (1) from a genome-wide DNA-microarray analysis of *Drosophila* NF-κB mutants following infection with the entomopathogenic fungus *Beauveria bassiana* (De Gregorio et al., 2002); (2) from DNA microarray studies of ectopic *Drosophila* NF-κB expression in larvae (Pal et al., 2008); (3) from a genome-wide RNAi screen in S2 *Drosophila* cells for gene products required for phagocytosis of *C. albicans* (Stroschein-Stevenson et al., 2006); and (4) from a DNA microarray analysis in *Drosophila* S2 cells following *C. albicans* infection (Levitin et al., 2007). All genes were tested with at least two different RNAi lines using the GAL4/UAS system (Brand and Perrimon, 1993). Gene expression was concomitantly depleted in three immunocompetent tissues: haemocytes (blood cells), fat body (the insect equivalent of adipose tissue) and the enterocytes (gut). Only six out of the top 24 targets identified have a previously documented role in immunity. To verify the relevance of *Drosophila* as a screening tool, we further analysed the gene encoding the α-(1,3)-fucosyltransferase FucTA (human homologue: FUT3), as fucosylation was found to be important in a mouse model of vaginal candidiasis (Hurd and Domino, 2004).

## RESULTS AND DISCUSSION

### Workflow and logic of the RNAi screen

**Fig. 1.**
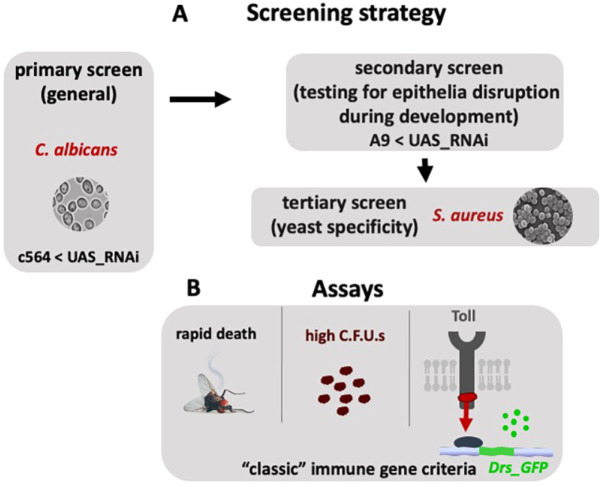
**Workflow of the screen.** (A) We expressed every UAS-RNAi in immunocompetent tissues (haemocytes, gut and fat body) through the *c564-GAL4* and infected flies with *C. albicans* or *S. aureus* to identify genes important for host survival following infection. Positive hits were tested for their ability to disrupt tissues during development by expressing the relevant UAS-RNAi lines in the developing wing via the *A9-GAL4*. (B) In addition to their survival following infection, positive hits were tested for pathogen growth by measuring colony forming units (CFUs) as well as the expression of the AMP *drosomycin* (*Drs*). The knockdown profile of a ‘classic’ immune gene in the Toll pathway (e.g. *Myd88* was used as a control) would be rapid death after infection, with an uncontrolled increase of CFUs and lack of *Drs* induction.

To identify new genes that are implicated in host survival following infection, we took the candidate gene approach. We wanted to address host survival following *C. albicans* infection when gene expression was depleted via RNAi. The overall depiction of the RNAi screen workflow is shown in Fig. 1. We used the library of the Vienna *Drosophila* Stock Centre (VDRC), which largely provides two kinds of stably integrated UAS-transgenic lines (Dietzl et al., 2007). Those where the transgene has randomly integrated via P-element transformation into the genome (GD lines) and those that have been integrated in the same engineered pChi31 site (KK lines). The latter are considered a more consistent alternative, as the integration site should not contribute to transcriptional variation due to the fact that all transgenes are targeted into the same genomic position. However, in addition to the originally intended 30D integration site, ∼25% of KK lines are also integrated into a second position (40D) due to meiotic recombination (Vissers et al., 2016). This causes false-positive phenotypes (Green et al., 2014; Vissers et al., 2016). Therefore, among other controls (such as prediction of no off targets), all final targets were tested by genomic PCR for the integrity of their single insertion at 30D and the absence of a transgene at 40D based on previously developed diagnostic PCR tests (Vissers et al., 2016).

For knocking down gene expression, we used the c564-GAL4 line, which has been shown to be expressed in three key immunocompetent tissues: the fat body (Zaidman-Rémy et al., 2006; Paredes et al., 2011), the haemocytes (Zaidman-Rémy et al., 2006) and the gut (Lajeunesse et al., 2010). When a line was susceptible at least twice in two independent infection experiments (carried out using flies from a subsequent generation and with a different batch of the same pathogen strain), we used another GD or KK line (if available) to verify the result. This way our emphasis was on repeat data to enhance reliability.

After determining the targets (see below), we crossed the RNAi lines with the A9 GAL4 driver, which is expressed in the developing wing epithelium from the early larval stage (Sun and Artavanis-Tsakonas, 1997). This was to ascertain whether depletion of these genes led to epithelial or tissue damage in at least one epithelium/tissue: the developing wing. This was not strictly a criterion for rejecting a target, but we believe it provides additional information on possible roles when combined with gene ontology terms.

### Defining and scoring screen hits

Genetic screens in which the defining metric for calling a hit is host survival are notoriously difficult. This is due to the variability that is inherent in assaying host survival multiple times across different genotypes. However, such screens provide the opportunity to obtain a holistic view of host defence in what is most important: what sustains a living host following an acute reaction to pathogenic infection.

In an effort to streamline our endeavour, we calibrated the pathogen dose on the flies we used as a ‘wild type’ control: line 25174 from the DGRP (Mackay et al., 2012). Microinjecting 300 cells of the *C. albicans* reference strain SC5314 consistently generated 50% survival at day 3 post-infection while injection of 100 cells of the *S. aureus* NCTC 8325-4 reference strain generated 50% survival at day 2 post infection. During the screen, susceptibility to infection was each time defined as those fly lines that succumbed rapidly after immune challenge relative to the population for all RNAi lines infected on the same day (using the same needle and same pathogen culture) and compared with the positive control c564GAL4; Myd88-RNAi flies, which succumbed in 24 h with infection whether they were infected with 300 cells of *C. albicans* or 100 cells of *S. aureus*.

**Fig. 2.**
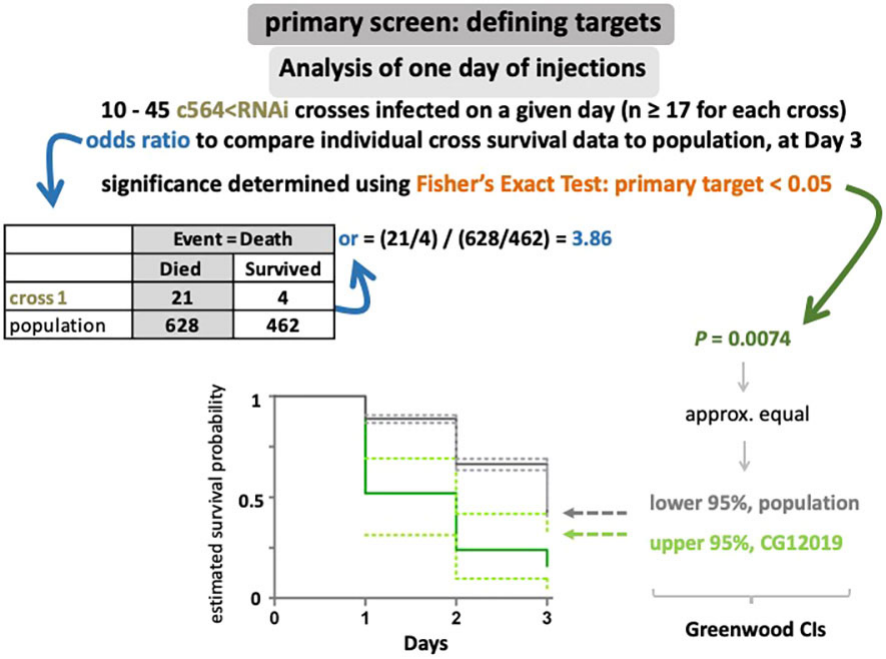
**Defining targets.** An odds ratio was used to compare the survival of each individual *c564<RNAi* cross to the infected population. As shown in the example, ‘cross 1’ was compared against the survival of all flies injected on that day. Comparisons were made for survival at day 3 post-infection and *P*-values were extracted using Fisher's exact test. Any *c564<RNAi* cross with *P*<0.05 was called a ‘primary target’. When primary targets were removed, all *c564<RNAi* crosses that were significantly different in their survival compared with the remaining population were ‘secondary targets’. Estimated survival probabilities were plotted according to Kaplan-Meir analysis, and Greenwood-type calculations of the 95% confidence intervals (CIs) for these rates were based on the standard errors of the primary estimates.

Infections were conducted as follows. Ten to 45 c564<RNAi crosses were microinjected on a given day (*n*≥17 for each cross). Odds ratio was used to compare the survival of each individual cross to the combined survival data of the population of flies injected that day. Comparisons were made for survival at day 3 post-infection and *P*-values were extracted using Fisher's exact test rather than the Log-Rank Test, as that would have been able to reveal more subtle but significant differences in the comparisons of individual crosses with the population when survival curves crossed. Any c564<RNAi crossed with a *P*<0.05 was called a ‘primary target’. When primary targets were removed, we ran the analysis again and all c564<RNAi crosses that were significantly different (*P*<0.05) in their survival compared with the population were deemed ‘secondary targets’. An example of the analysis for one day of infections is shown in Fig. 2.

**Fig. 3.**
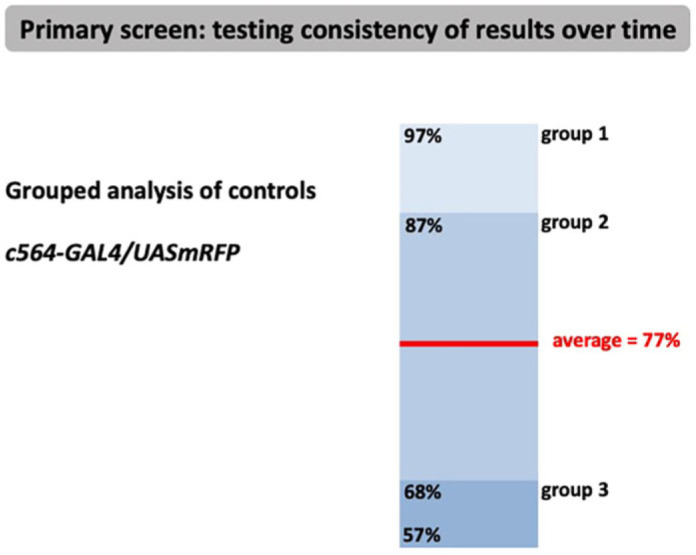
**Control independent of the RNAi mechanism.** To avoid potential information loss due to differences between stronger or weaker pathogen cultures used on different experimental days, we employed an internal *c564-GAL4<UAS-mRFP* control. This was useful to compare the survival of each group on a particular day versus the control, as well as comparing datasets across the entire screen. Grouped datasets of *c564-GAL4<UAS-mRFP* controls were analysed as for day of injection data (shown in Fig. 2).

To mitigate the potential variability of the survival assay and also include a control independent of the RNAi mechanism, we also infected c564-GAL4<UAS-mRFP flies. Thus, at the start and end of each day of infection, we injected a c564-GAL4<UAS-mRFP cross. This was to see the consistency of controls over time during the same day and also to compare globally the consistency of controls over the whole period of the screen. The problem we wanted to address was twofold. First, on some days, datasets were small (a few RNAi lines injected) whereas on other days, datasets included many more RNAi lines. Second, we needed to be able to compare days where injections were overall very strong with a uniformly reduced survival. This could be mitigated for the day by comparing each individual line with the survival of the overall population for that day, but it would be difficult to compare data across days. We therefore introduced data groupings based on survival data for an internal UAS-mRFP and the control-grouped datasets were analysed as for day of injection data. The spread of values of the controls themselves over the course of the screen, as well as the spread of survival of the RNAi compared with their daily *c564-GAL4<UAS-mRFP* control, enabled us to test the consistency of our results over the time (Fig. 3). Thus, in addition to the population survival data of their day, each line was also grouped with regards to the survival of the internal UAS-mRFP control that was carried out on the same day. Thus, we were able to circumvent the problem of potential information loss when comparing across datasets from different days (Fig. 3).

For *C. albicans* infection, over the course of the screen, we identified 239 KK and 152 GD lines as primary susceptible targets, and 139 KK lines and 131 GD lines as secondary susceptible targets. However, there was an overlap between primary and secondary targets as 37 KK lines and 30 GD lines were scored as primary and secondary targets in one of the two biological repeats. In that case, we repeated the infection at least a third time and/or used an additional RNAi line (when available) to finally refer to the target as primary, secondary or non-target. All results for *C. albicans* infection survival screening are contained in Table S1.

From the targets identified through *C. albicans* infection, nine out of 152 GD lines were also scored as primary targets and four out of 131 GD lines were also scored as secondary targets with *S. aureus* infection. For KK lines, the relevant numbers were: 29 out of 239 KK lines also scored as primary targets and 10 out of 139 KK lines also scored as secondary targets. Table S2 contains all lines that were initially selected through susceptibility to *C. albicans* but were also scored as positive hits after *S. aureus* infection. This meant that there was an important distinction in the genes underscoring host survival between *C. albicans* and *S. aureus* infection.

### RNAi leading to compromised host survival following *C. albicans* infection

**Fig. 4.**
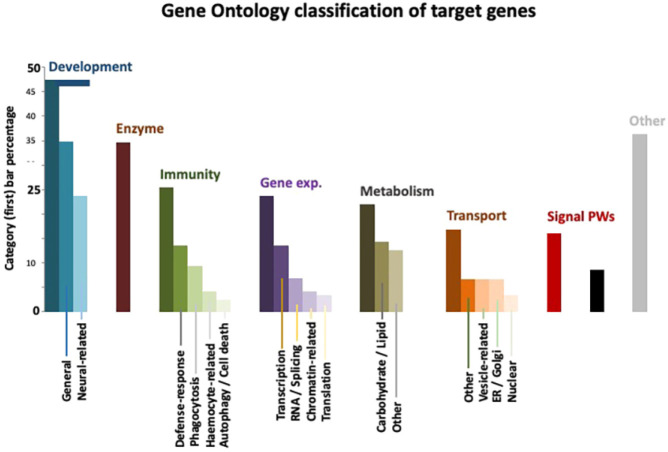
**Gene ontology classification of targets.** Category (first) bar gives percentage of genes in that category relative to all targets. Inner bars give the percentage of genes relative to the category bar.

Our screen has uncovered new regulators of host survival following *C. albicans* infection. Using Gene Ontology (GO) definitions, these genes were categorized as having a role in development, enzymatic regulation, immunity, gene expression, metabolism, and transport (Fig. 4). A number of these are *Drosophila* homologues of human genes not previously implicated in host survival after this immune challenge, although two targets have been already established in host resistance to *C. albicans*: Ferritin (Potrykus et al., 2013) and EGFR (Ho et al., 2019). A selection of the most consistent *C. albicans-*specific gene knockdowns (top targets) is shown in Table 1. Data on the different RNAi lines used to screen all top targets (including their survival at day 3 following *C. albicans* infection and A9 data) can be found in Table S3.

**Table 1.**
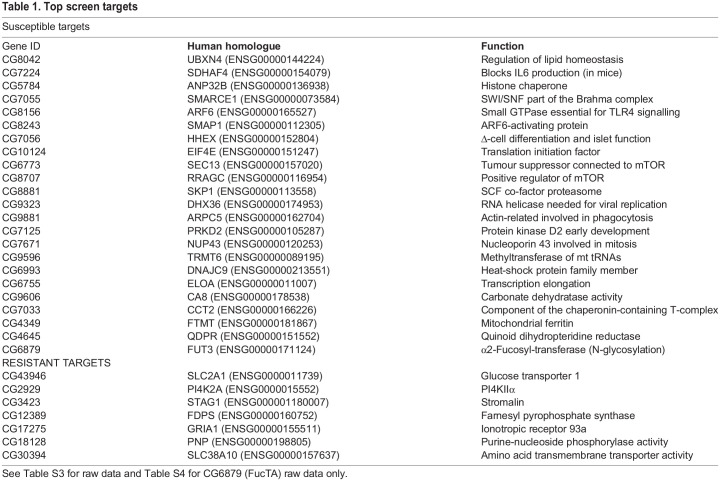
Top screen targets

*C. albicans*-specific targets implicated in gene expression include CG11006, which is involved in the regulation of transcription (human homologue SAP130); CG6843, a co-repressor (along with RBPJ) of CBF1 (human homologue CIR1); CG10228, which is implicated in mRNA cleavage and polyadenylation (human homologue PCF11); and CG1109, which is involved in mRNA 3′ processing (human homologue WD R33). Several are predicted or have been shown to have signalling capacity (such as CG12737, which encodes a GDP/GTP exchange factor for Rab10 and Rab11), while the protein encoded by *arfgap2* (human homologue SMAP2) is a GTPase activator. Several have enzymatic activity, such as the carbonate dehydrogenase CG6906 (human homologue CA1), the SCF complex component SKP1, the lipase CG10116 (human homologue LIP1), the CG4665 short-chain dehydrogenase (human homologue QDPR) and the DEAH-box helicase CG9323. We also found 88 RNAi that survived significantly better than the control. In the context of the screen, this meant >70% of flies alive after 72 h. To validate this, we increased concentration to four times the amount of pathogen we were using for the original screen [a total of ∼1200 (4×300) cells per injection] and from the 88 RNAi lines, seven KK lines were consistently surviving significantly better than the population average and the UAS-RFP control. These seven genes are also shown in Table 1. It is interesting to note that most of these genes encode proteins involved in metabolism and especially transport of metabolites across membranes. More work on fungal load is needed to ascertain whether this is resistance to the pathogen (host survival due to low fungal load) or tolerance without impacting on pathogen fitness (potential high fungal load).

### Susceptibility to *C. albicans* reveals a role for protein fucosylation in host survival

From the RNAi lines that were highly susceptible to both pathogens used in the screen, one was against FucTA, an alpha1,3-fucosyltransferase. FucTA encodes a Golgi fucosyltransferase that transfers fucose in α1,3-linkage to core N-acetylglucosamine residues of N-linked oligosaccharides. In *Drosophila*, haemocyte-specific N-linked glycosylation is required for encapsulation of foreign bodies (Mortimer et al., 2012), while in humans, TLR4 requires N-glycosylation for signalling through MD2 (da Silva Correia and Ulevitch, 2002). A common null mutation within the coding region of the α-(1,2)-fucosyltransferase gene, *FUT2* (secretor factor gene), leads to ABO and Lewis histo-blood group antigen non-secretion from mucosal tissues in ∼20% of humans (Koda et al., 2001). Non-secretor status has been associated with differences in susceptibility to several viral and bacterial infections, including *C. albicans* (Chaim et al., 1997). Based on *in vitro* studies and on a mouse vaginal candidiasis model, a host-microbe adhesion mechanism has been proposed (Hurd and Domino, 2004). In addition, in humans, sequence variations in *FUT3* impact the solubility and stability of Lewis antigens, as FUT3 is required for the last step of their synthesis (Kukowska-Latallo et al., 1990).

The major pathway for N-glycan fucosylation in *Drosophila* has been described in the embryo (reviewed by North et al., 2006). The pathway begins with the biosynthesis of a dolichol-linked precursor molecule that is then transferred to an Asn residue in the target protein by oligosaccharyltransferase (Ost). The precursor molecule is then trimmed by the activity of the α-glucosidase enzymes to produce oligomannose-type N-glycans. The latter process is initiated by the addition of fucose (Fuc) in α6-linkage to NM3N2 (MGn) by Fucosyltransferase 6 (FucT^6^), followed by addition of α3-linked Fuc, which is catalysed by FucTA. The latter ‘prefers’ substrates already containing α6-linked Fuc; thus, most α3-fucosylated glycans are difucosylated. Further processing is then provided by the GlcNAc transferase Mgat1 (Sarkar et al., 2006).

**Fig. 5.**
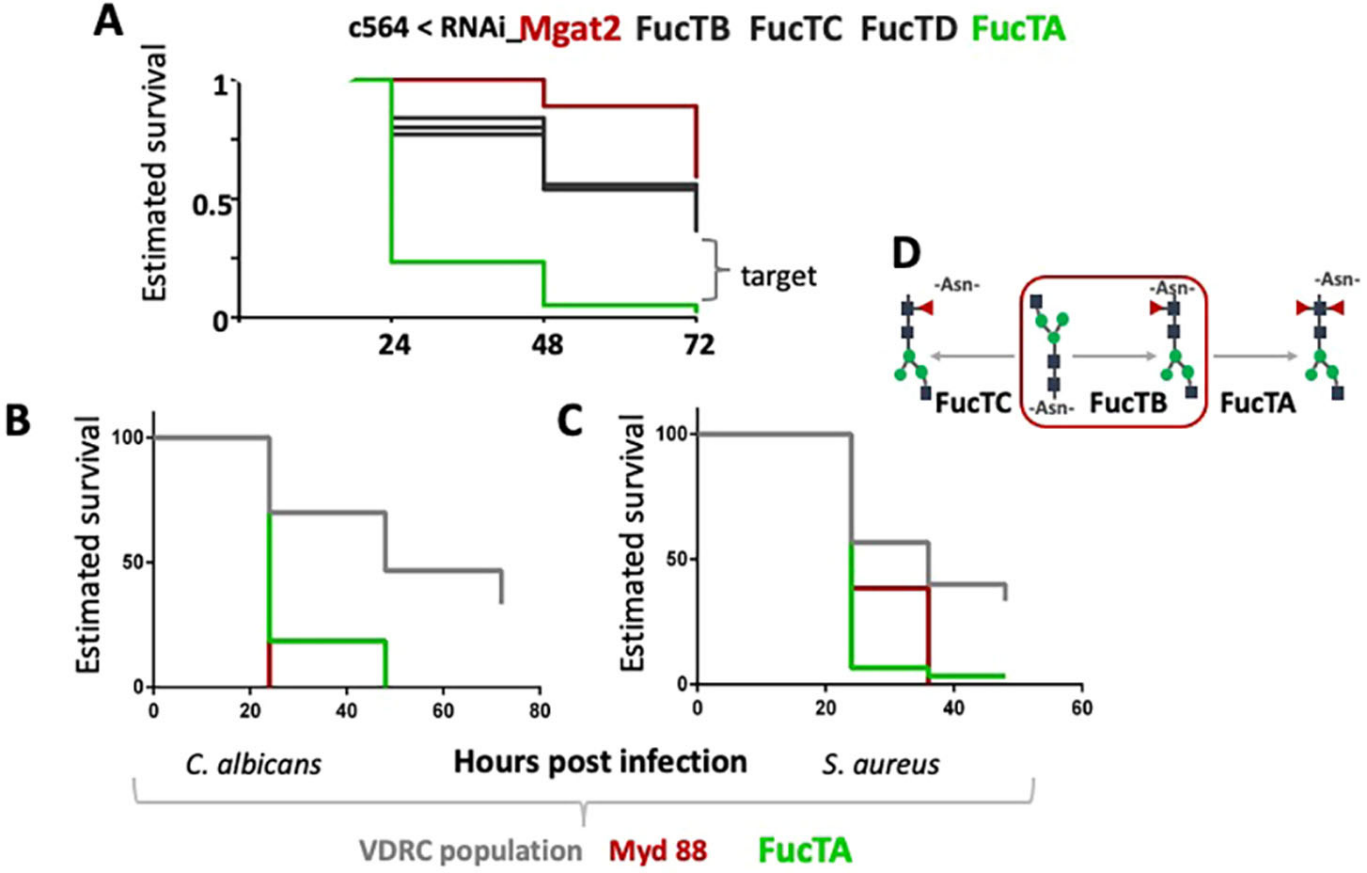
**FucTA is needed for *Drosophila* to survive *C. albicans* and *S. aureus* infection.** (A) FucTA but not FucTB, TC, TD or Mgat2 was needed for survival following *C. albicans* infection. (B,C) FucTA RNAi flies were susceptible to (B) *C. albicans* and (C) *S. aureus* infection*.* (D) The N-glycan pathway in *D. melanogaster*. Production of oligomannose-type N-glycans is initiated by the addition of fucose (Fuc) in α6-linkage to NM3N2 (MGn) by Fucosyltransferase 6 (FucTB), followed by addition of α3-linked Fuc, which is catalysed by FucTA. The latter ‘prefers’ substrates already containing α6-linked Fuc; thus, most α3-fucosylated glycans are difucosylated.

From the enzymes needed for the different modifications in the *Drosophila* N-glycan pathway, only FucTA-deficient flies were susceptible to *C. albicans* infection (Fig. 5A). As mentioned above, RNAi-mediated FucTA knockdown produced flies compromised in their survival after both *C. albicans* (Fig. 5B) and *S. aureus* infection (Fig. 5C). Of note, a characteristic of FucTA is that it produces the difucosylated *N*-glycans (Fig. 5D) recognized by anti-horseradish peroxidase (anti-HRP) antisera, providing a well-established marker for insect neural tissue (Fabini et al., 2001).

**Fig. 6.**
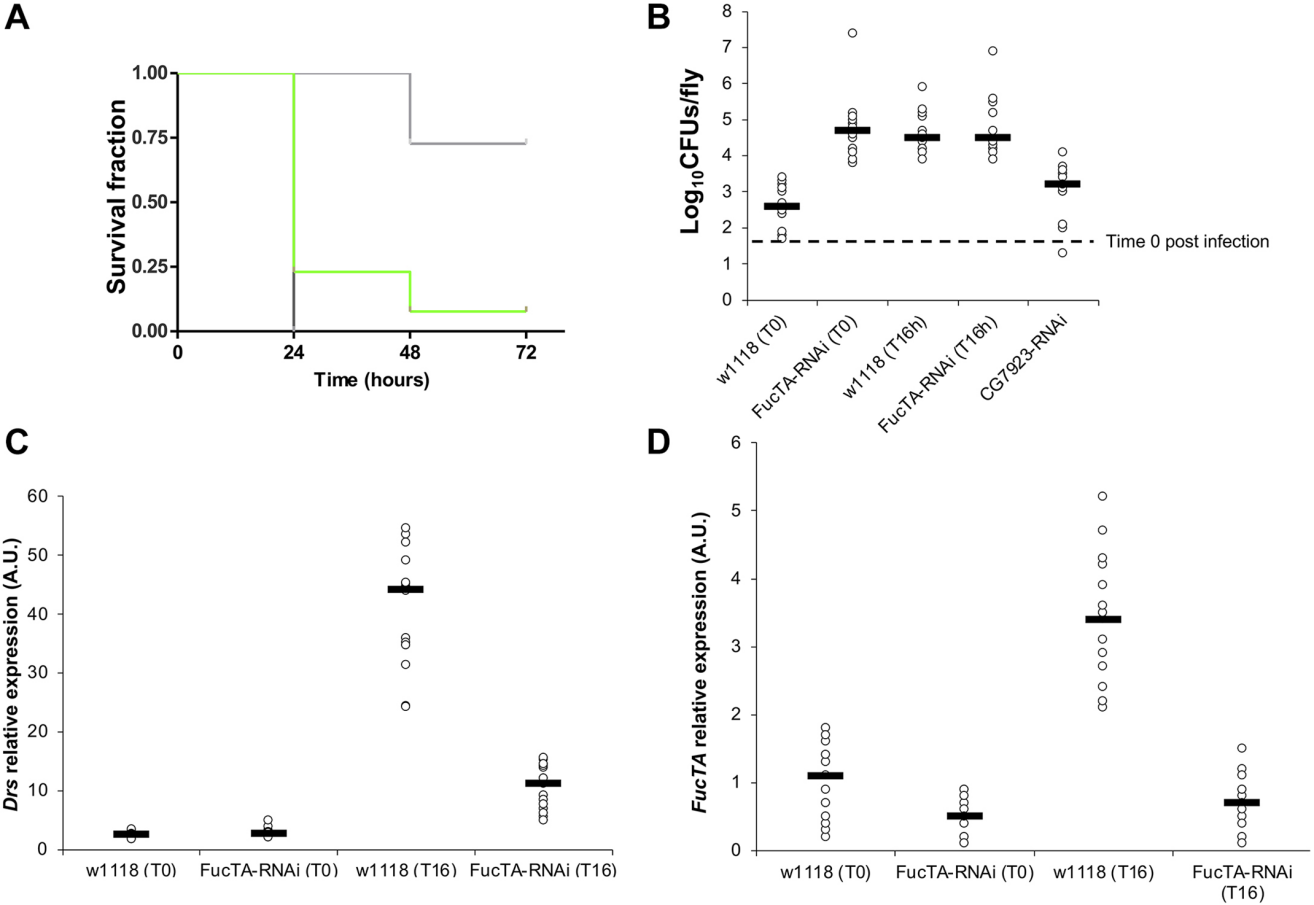
***Drosophila* depleted from FucTA are susceptible to infection.** (A) Survival of *c564-GAL4<UAS-FucTA^RNAi^* following *C. albicans* infection was statistically indistinguishable from *c564-GAL4<UAS-Myd88^RNAi^* at the LT_50_ point (*P*>0.1, log-rank test). Both were statistically different from the negative control *c564-GAL4<UAS-CG7923^RNAi^* (*P*<0.005, log-rank test). The latter was used as a non-target to ascertain that the RNAi mechanism was not responsible for the susceptibility to infection. (B) Pathogen growth immediately following infection was significantly higher (*P*<0.005) in *c564-GAL4<UAS-FucTA^RNAi^* compared with the *w^1118^* control. CFUs in the latter increased as the infection progressed and were indistinguishable from *c564-GAL4<UAS-FucTA^RNAi^* at 16 h post-infection. (C) *Drs* expression of *c564-GAL4<UAS-FucTA^RNAi^* was significantly reduced compared with the VDRC *w^1118^* genetic background. (D) Of note, transcription of *FucTA* was induced following *C. albicans* infection in *w^1118^* but not in *c564-GAL4<UAS-FucTA^RNAi^* flies. For all scatterplots, each dot is one fly; for every genotype or treatment, *n*=15. Black lines represent each the median value of each group. T0 is the point immediately after infection (realistically, 50 min after injecting the last fly) and T16 is the point 16 h after infection. At each time point, the data followed a normal distribution with equal variance; one-way ANOVA was therefore used to look for differences. Following infection, 95% Tukey HSD intervals revealed significant differences between the *w^1118^* background and the *FucTA* RNAi in all assays.

The raw survival data are included in Table S4. FucTA knockdown caused reduced survival following *C. albicans* infection that was comparable with Toll RNAi (Fig. 6A). When assayed immediately after infection, in the absence of FucTA, *C albicans* CFUs were significantly higher than control flies (Fig. 6B). As infection progressed, FucTA-RNAi CFUs remained high but were statistically indistinguishable from the control at 16 h post-infection (Fig. 6B). Expression of the antifungal peptide gene *drosomycin* was significantly reduced (Fig. 6C) when FucTA expression was reduced (Fig. 6D). Of note, in *w^1118^* flies, *C. albicans* infection induced gene expression of *FucTA* (Fig. 6D). Susceptibility to infection was confirmed using flies carrying a mutant allele (*FucTA^f03774^*) of FucTA (Fig. S1A), a phenotype that was reversed when a precise excision of the transposable element responsible for the mutation was obtained (Fig. S1B, FucTA ‘rescue’). This meant that depletion of FucTA had the ‘classic’ immune gene phenotype: (1) reduced host survival, (2) increased pathogen growth and (3) low AMP response. Finally, *FucTA* transcription was also induced by *S. aureus* infection (Fig. S2), a result compatible with the well-conserved NF-κB sites present in its promoter.

### Using the HRP epitope to find fucosylated proteins that influence survival of infected flies

**Fig. 7.**
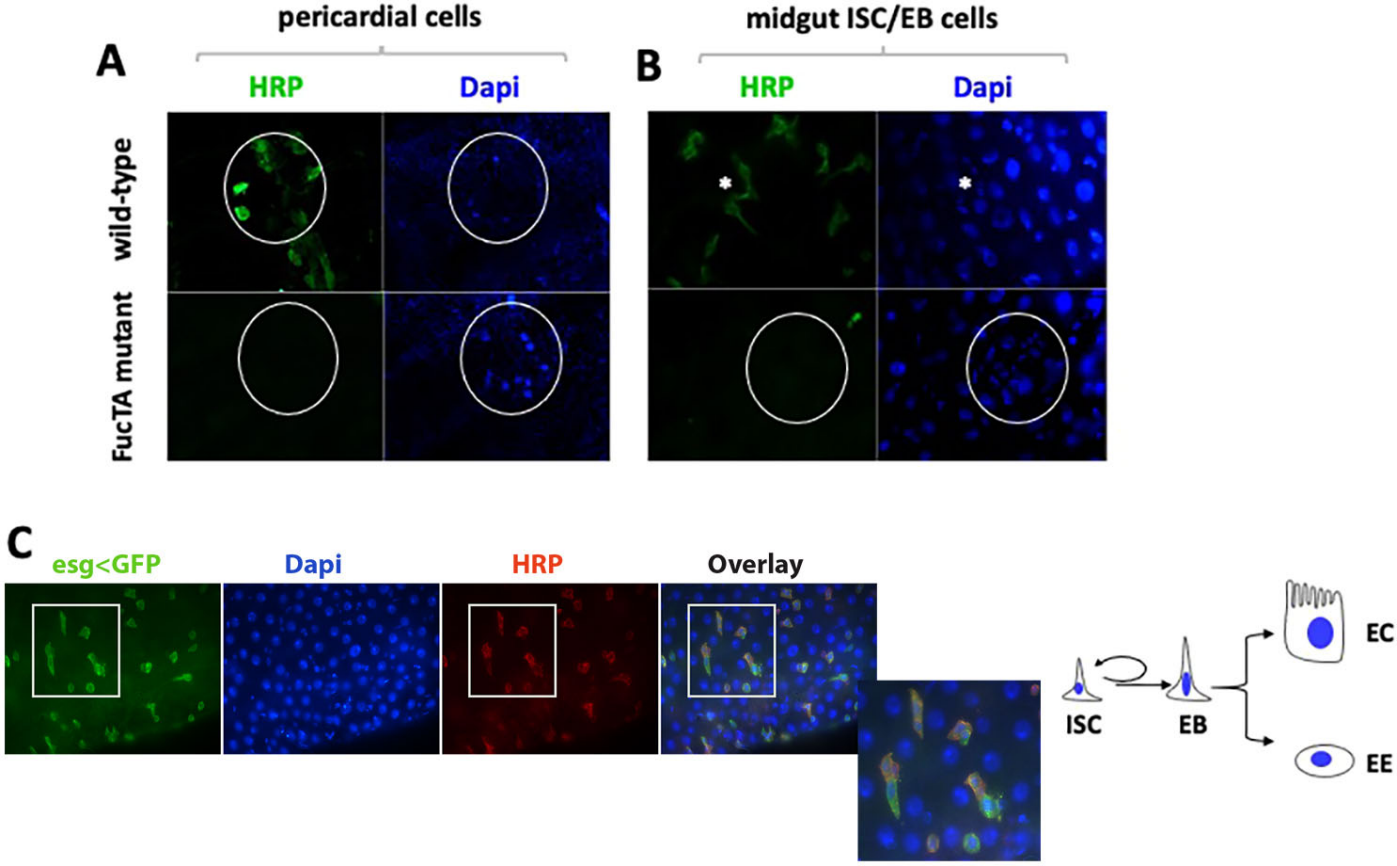
**The FucTA-dependent HRP epitope is detected in pericardial and midgut progenitors.** (A,B) In *FucTA^f03774^* mutant flies, the HRP epitope is abolished in (A) pericardial cells and (B) midgut intestinal progenitors. (C) During intestinal progenitor cell division, the HRP epitope decorates intestinal stem cells (ISCs, smaller cells) but not enteroblasts (EBs, larger more elongated cells). As the schematic shows, after EB production from ISC division, EBs will differentiate without cell division to enterocytes (ECs) or (less often) to enteroendocrine cells (EEs).

We used the difucosylated *N*-glycans recognized by the anti-HRP antibody for affinity purification coupled to high-resolution mass spectrometry to isolate FucTA-modified HRP glycoproteins. First, we verified that FucTA is responsible for the addition of that epitope, as *FucTA^f03774^* flies were negative for HRP staining in the three tissues where, according to the Fly Atlas, FucTA gene is scoring the highest expression: the heart (pericardial cells, Fig. 7A) and the gut (Fig. 7B,C). Using the anti-HRP antibody, we then compared the HRP glycoproteins appearing in the eluate of wild-type flies injected with a sterile saline (PBS) buffer to those infected with *C. albicans* (8A) compared with appropriate controls for buffers and elution targeting non-HRP epitopes (8B). Mass spectroscopy of the eluates showed a number of proteins modified with the HRP epitopes in increased quantities following infection (Table 2).

**Table 2.**
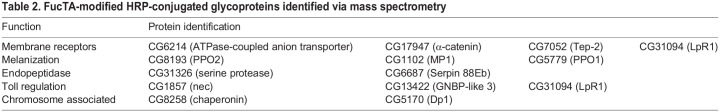
FucTA-modified HRP-conjugated glycoproteins identified via mass spectrometry

Included in these proteins was one of the six *Drosophila* orthologues of the mammalian LDL Receptor family, LpR1. In a *FucTA^f03774^* mutant background, LpR1 was stabilized compared with wild type with and without infection (9A). Lpr1 is the receptor for the serine protease inhibitor Necrotic (Nec) and its target protease (Soukup et al., 2009). Nec clearance is extremely rapid, but deletion of the *LpR1* gene sensitizes the immune response: *nec* transcript levels decrease and *Drs* transcript levels increase (Soukup et al., 2009). This implies a regulatory feedback loop at the transcriptional level. In this context, it is significant that LpR1 appears to bind the non-inhibitory serpin/proteinase complex, in preference to the native Nec serpin. Thus, clearance of the serpin/protease complex appears to compete with a regulatory feedback loop affecting *nec* transcription. Conversely, stabilization and increase of LpR1 (and Nec, see Table 2) levels in the *FucTA^f03774^* mutant would decrease *Drs* gene expression (as we show in Fig. 6C), which would underscore the *FucTA^f03774^* susceptibility to infection.

**Fig. 8.**
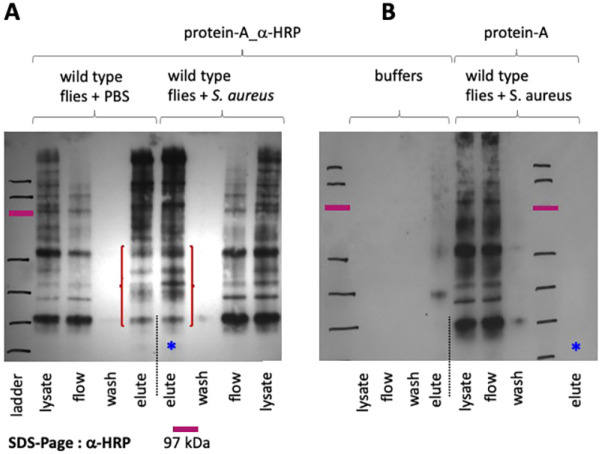
**Using the HRP epitope to identify targets of FucTA-mediated fucosylation after infection.** (A) Western blot analysis of HRP pulldown assays on whole-cell lysate from flies infected with *S. aureus* or injected with PBS. All wash and elution stages are indicated. (B) Shown are the negative control for buffer, i.e. assay as in A but only using buffer, or non-specific binding, i.e. using magnetic protein beads alone. Protein ladder (indicated by black/red marker on film) was marked on the film by superimposing the film on the membrane. The top bar of the protein marker ladder in A and B has a molecular mass of 190 kD.

**Fig. 9.**
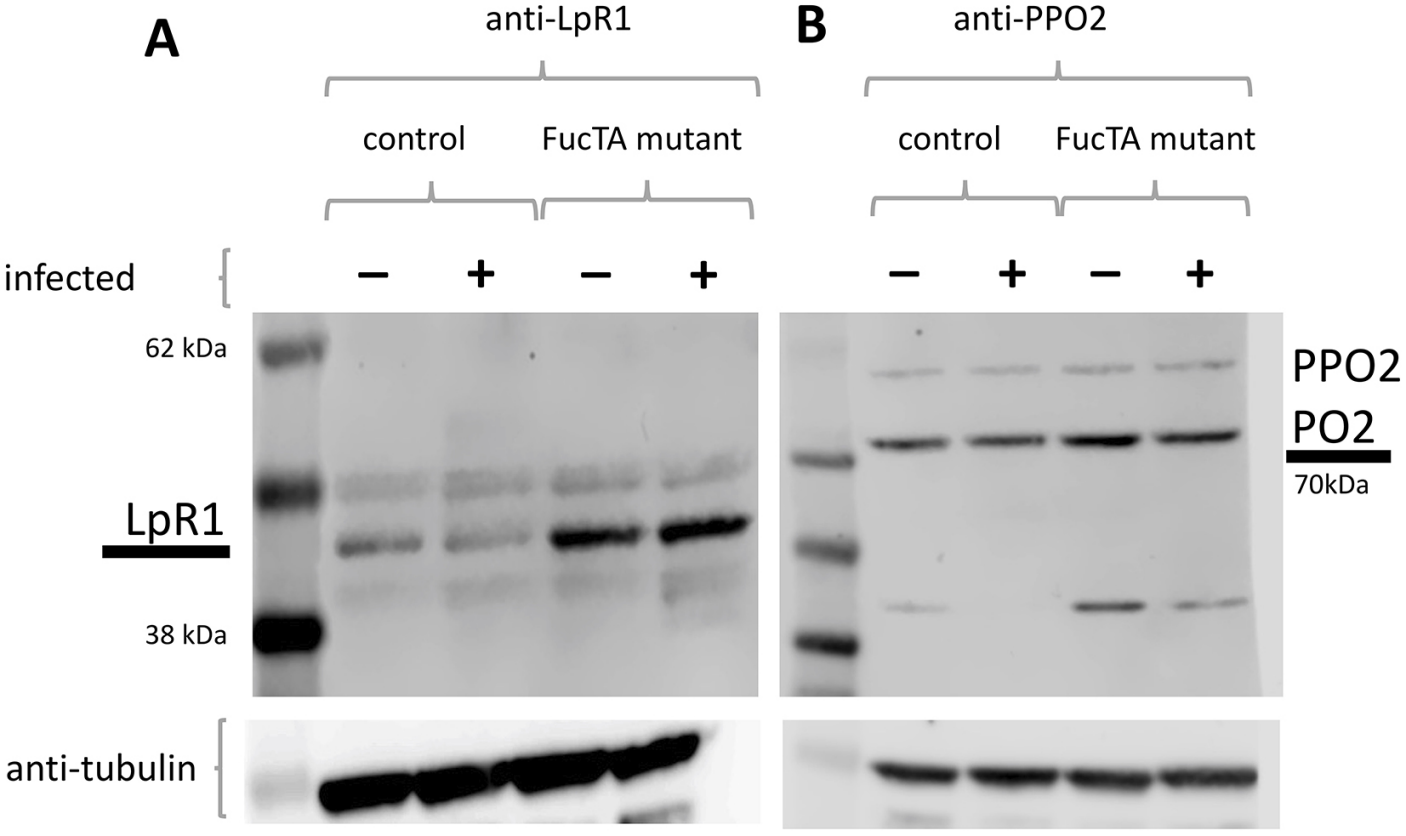
**Protein levels of LpR1 and PPO2 increase in the absence of fucosylation.** (A) Loss of FucTA results in higher levels of LpR1 independently of infection. (B) Loss of FucTA results in higher levels of processed PPO2 (PO2) that are reduced following infection (as in control *yw* flies) but that are still higher in *FucTA^f03774^* infected flies than in control infected flies.

An additional protein found to be increased in *FucTA^f03774^* was ProPhenolOxidase 2 (PPO2) (Fig. 9B). PPO2 is important for the melanization reaction, a major immune response in arthropods (reviewed by Cerenius et al., 2008). It involves the rapid synthesis of a black pigment, melanin, at the site of infection and injury. Melanization requires the activation of PPO2, an enzyme catalysing the oxidation of phenols to quinones, which polymerize to melanin. PPO2 has been shown to be the major component stored and able to be released following wounding or infection after the immediate acute phase (Binggeli et al., 2014). Six hours after *C. albicans* infection of wild-type flies, PPO2 is undetectable (Fig. 9B). In flies lacking fucosylation (*FucTA^f03774^*), PPO2 is increased without infection and reduced but not used up as it is in wild-type flies following immune challenge (Fig. 9B). This indicates a reduced melanization reaction, as shown in direct measurements of melanization in haemolymph (Fig. S3). In turn, this would explain the susceptibility to infection of FucTA-deficient flies.

### New genes involved in host survival to *C. albicans* infection

Altogether, our results highlight multiple genes in a variety of functional classes that influence host survival following *C. albicans* infection specifically or host survival following both *C. albicans* and *S. aureus* challenge. Many of these genes have strong human homologues and, given the evolutionary conservation of innate immune responses needed for flies and mice to fight off *C. albicans,* they provide avenues to consider in deciphering the basic biology of host survival to infection.

Fucosylation has been firmly established to have diverse roles in the mammalian immune system (reviewed by Li et al., 2018). These include its role in polarization and function of M1 macrophages where expression of TNF strongly correlates with expression all FUT genes (*FUT1* to *FUT12*) (Li et al., 2014), the fucosylation of the μ heavy chain in B-cell development in the bone marrow (Li et al., 2014), the modulation of TCR interaction with MHC-antigen complexes (Field et al., 2016), the fucosylation of IgGs (Burton and Dwek, 2006) and the fucosylation induced by commensal bacteria (Pickard et al., 2014). N-glycan fucosylation has also been specifically implicated in host resistance to *C. albicans* and *S. aureus* infections (Hurd and Domino, 2004). This is the first time that α1,3-linkage of N-linked oligosaccharides has been implicated in *Drosophila* immune defence. This indicates a potential evolutionary conservation and identifies a need for fucosylation in survival of both flies and mammalian hosts after infection.

## MATERIALS AND METHODS

### Fly stocks

All UAS-RNAi transgenic fly lines (KK and GD) were obtained from the VDRC. A *UAS-RFP* line was used as a transcriptional reporter to verify efficiency of the UAS/Gal4 expression system and as control for fly survival. The GAL4 drivers fly lines *c564-GAL4*, *yolk-GAL4* and *A9-GAL4* as well as the *FucTA^f03774^* mutant strain used in the study were obtained from Bloomington *Drosophila* Stock Centre (BDSC). A precise excision of the *FucTA^f03774^* insertion (*FucTA* ‘rescue’ see Fig. S2) was performed using the *yw*, Δ2-3 stock from BDSC.

### Microbial strains

We used the *C. albicans* strain SC5314 (Jones et al., 2004), stored in 25% glycerol at −80°C, and routinely grown on Sabouraud's agar (SGA) and Sabouraud's broth (SGB). We also used the *S. aureus* reference strain NCTC 8532 (Public Health England, Culture Collections).

### Infection

To infect flies, the *Candida albicans* (*C. albicans*) strain was cultured in SGB (Oxoid) for 18 h; cells were harvested by centrifugation (694 ***g*** for 5 min) and washed in sterile phosphate-buffered saline (PBS). Washed fungal cells were again centrifuged and re-suspended in PBS to an optical density of ∼0.95-1.05 (Thermo Scientific NanoDrop 1000 spectrophotometer). The inoculant containing *C. albicans* strain was further diluted fourfold in PBS. Similarly, *Staphylococcus aureus* (*S. aureus*) NCTC8325-4 was cultured in TSB for 16 h; cells were harvested by centrifugation (868 ***g*** for 7 min) and washed in PBS. Cells were then centrifuged and re-suspended in PBS to an optical density of approximately 0.360 and further diluted 1000-fold in PBS for injection. Anaesthetized female flies were infected with 13.2 nl of the *C. albicans* or *S. aureus* suspensions (or with PBS control), directly injected into the haemolymph through the dorsolateral region of the thorax, using a micro-injector (Drummond Scientific Nanoinject II). The number of viable yeast cells injected per fly was ∼600, as calculated from plating homogenates of six injected flies, previously ground in SGB medium. Flies were kept at 30°C post-infection for 36 h and then dissected.

### Primary screen

Transgenic UAS-RNAi (KK and GD lines) males were crossed to virgin females of a GAL4 driver expressed in immunocompetent tissues, i.e. fat body, haemocytes and gut (*c564-GAL4*), or to virgin females of a specific wing driver (*A9-GAL4*). The latter was to check for non-inflated wings, which is an indication of non-specific phenotypes (Green et al., 2014). At least two independent UAS-RNAi constructs (KK and GD), when available, were tested to assess the targeting potential of each construct. After 13 days at 25°C, the viable F1 offspring was transferred to 30°C in order to maximize GAL4 activity and thus the RNAi expression and consequent repression of the target genes before injection. On the 15th day, progeny female flies were injected with 10 nl of *C. albicans* or *S. aureus* inoculant directly into the haemolymph (via thorax) using a nano-injector (Nanoject II, Drummond Scientific). Following injection, flies were stored at 30°C and assessed for survival every 24 h (*C. albicans*) or every 12 h (*S. aureus*) over the course of 3 days.

### Dissection and tissue immunostaining

For gut and pericardial cell imaging, anesthetized flies were dissected in Schneider's medium and fixed for 30 min in 4% paraformaldehyde (in PBS), rinsed in PBS and then washed three times (5 min each) in wash solution: 0.1% Triton X-100 (Sigma-Aldrich) in PBS. The tissue was blocked for 60 min in blocking solution [0.1% Triton X-100 and 2% BSA (Sigma-Aldrich) in PBS] and immunostained with primary antibodies overnight at 4°C. Samples were then washed four times for 5 min each at room temperature in wash solution, incubated with secondary antibodies at room temperature for 2 h, washed again as before and them stained with DAPI (1:1000, Sigma-Aldrich). Washed tissues were mounted in slides with Vectorshield mounting media (Vector Laboratories). The primary antibody goat anti-HRP (123-165-021, Jackson ImmunoResearch Labs) at 1:500 was used with the secondary antibody donkey anti-goat Alexa 568 (Invitrogen; 1:250).

### Colony forming units (CFUs) determination

CFUs were determined at three different time points (0, 14 and 36 h) using six female flies from the F1 progeny of the *c564-GAL4* cross with UAS-RNAi lines. Flies were homogenized, diluted serially and plated onto SGB agar medium and grown for 24 h at 37°C.

### Characterization of KK-RNAi lines

Genome landing sites of the KK-UAS-RNAi constructs for the candidate immune genes were analysed by PCR, according to Green et al. (2014) and wing characterization of F1 progeny obtained by crossing virgins of *A9-GAL4* with the VDRC UAS RNAi males.

### RNAi target analysis and Drs quantification by quantitative PCR (qPCR)

UAS-RNAi lines were expressed under the control of the *c564-GAL4* driver. Total RNAs were extracted from six female flies using the Total RNA Extraction Kit (Norgen) according to the manufacturer's instructions. Total RNA (500 ng) was used as a template for reverse cDNA transcription (SensiFast cDNA synthesis Kit, Bioline). Quantitative PCR reactions (SensiFast SYBR No-ROX Kit, Bioline) were carried out using 2 µl of cDNA template tenfold diluted and 400 nM of each primer, i.e. *Drs* (+) 5′-GTACTTGTTCGCCCTCTTCG-3′ and *Drs* (–) 5′-TTAGCATCCTTCGCACCAG-3′. The housekeeping gene *tbp* (Matta et al., 2011) was used as a control to normalize expression of the gene of interest. qPCR reactions were performed as outlined in manufacturer's instructions, amplicon amplification was carried out as 40 cycles of 5 s at 95°C, 10 s at 62°C and 20 s at 72°C. Each reaction was performed in duplicate in a Quiagen Rotor-Gene Q real-timePCR cycler with a 72-well rotor. mRNA levels were calculated with the comparative CT (threshold concentration) method.

### Data analysis

For all infections carried out with the same needle by the same person on the same day comparisons were made for survival at day 3 post-infection and *P*-values were calculated using Fisher's exact test. Each line was compared with the overall survival of all lines in the same injection cohort, as well as with *c564-GAL4<UAS-MyD88^RNAi^* and *c564-GAL4<UAS-RFP* (see below). Any *c564<RNAi* cross with *P*<0.05 was called a ‘primary target’. When primary targets were removed, we ran the analysis again and all *c564<RNAi* crosses that were significantly different in their survival compared with the population (minus primary targets) were considered ‘secondary targets’.

To mitigate the potential variability of the survival assay and include a control independent of the RNAi mechanism, we also infected *c564-GAL4<UAS-mRFP* flies. Thus, at the start and end of each day of infections, we injected a *c564-GAL4<UAS-mRFP* cross. This was to see the consistency of controls over time during the day but also to compare globally the consistency of controls over the whole period of the screen.

Thus, we introduced data groupings based on survival data for an internal UAS-mRFP, and the control-grouped datasets were analysed as for the day of injection data. The spread of values of the controls themselves over the course of the screen as well as the spread of survival of the RNAi compared with their daily *c564-GAL4<UAS-mRFP* control enabled us to test the consistency of our results over time.

### Infection and sample preparation for western blot analysis of LRP1 and PPO2

Overnight 10 ml cultures of *S. aureus* (NCTC8325-4) bacteria were washed and resuspended in an equal volume of sterile PBS, and further diluted 1/1000. Adult female flies from FuctA mutant or the isogenic background flies, 2-4 days old, were injected in the thorax with 10 nl of a bacterial cell suspension or PBS using a nanoinjector (Nanoject II, Drummond Scientific). For determination of CFUs, injected flies (six females) were crushed immediately in media appropriate for the bacteria injected and the homogenates were diluted and plated on tryptic soy agar-media (TSA). The plates were incubated at 30°C for 20-30 h and the CFUs per fly were measured by counting the number of colonies on each plate, the CFUs per fly were used to adjust the initial dose of bacteria injected to ∼100 CFUs per fly. 16 h after injection, flies (*n*=10) were homogenized in 250 µl RIPA buffer (Sigma-Aldrich) containing protease inhibitors. Protein concentrations present in the supernatants were determined using BCA protein assay (Thermo Fisher Scientific) and further adjusted with RIPA. After boiling the protein samples in protein sample buffer (Invitrogen) for 5 min, 18 µl of each sample and 6 µl of protein standard (LC5925 SeeBlue Plus2) were loaded onto a gel (NuPAGE Bis-Tris, Invitrogen) for SDS-PAGE.

### Western blot assays

Following electrophoresis, proteins were transferred to nitrocellulose or to polyvinylidene fluoride (PVDF) (Bio-Rad) membranes using the XCell II Blot Module (Invitrogen) according to the manufacturer's instructions. Western blotting was performed using standard protocols. Visualization of reactive proteins was performed by enhanced chemiluminescence and quantitative infrared imaging (LI-COR Odyssey, LI-COR Biosciences).

### Primary antibodies used in the study

Primary antibodies used were mouse anti-tubulin (1:2500, Sigma-Aldrich, catalogue no. T8328), rabbit anti-HRP (1:1000, Jackson ImmunoResearch, catalogue no. 123-165-021), rabbit anti-PPO2 (1:2000; Binggeli et al., 2014), guinea pig anti-LRP1 (1:200; Soukup et al., 2009). Secondary antibodies used were IRDye 800CW donkey anti-rabbit IgG (1:5000, Licor, catalogue no. 926-32213), IRDye 800CW donkey anti-guinea pig IgG (1:5000, Licor, catalogue no. 926-32411), IRDye 800CW donkey anti-rabbit pig IgG (1:5000, Licor, catalogue no. 925-32213) and IRDye 680CW donkey anti-guinea pig IgG (1:5000, Licor, catalogue no. 926-68077).

### Whole-fly protein extract and mass spectroscopy

Whole-fly protein extracts from PBS-injected or *C. albicans* infected were prepared following a modified version of a previously established protocol (Auluck et al., 2005). Ten 5-day-old flies for each category of genotype/treatment were homogenized in 70 μl of extraction buffer [20 mM Tris (pH 7.6), 50 mM NaCl, 1% Triton X-100 and protease inhibitor (Amresco)], vortexed gently and incubated on ice for 30 min. After centrifugation for 60 min at 15,000 ***g*** at 4°C, supernatants were collected and mixed with 4×LDS Sample Buffer and DTT containing (10×) Sample Reducing Agent (Life Technologies). The remaining pellets were resuspended in SDS extraction buffer [50 mM Tris (pH 7.6), 5 mM EDTA and 4% SDS], vortexed and boiled for 10 min. Supernatants were collected after centrifugation 10 min at 15,000 ***g*** and mixed with 4×LDS sample buffer and DTT as described above. Both fractions were boiled for 20 min before electrophoresis. For each extract, a volume corresponding to two flies was resolved on NuPAGE Novex 4-12% Bis-Tris Protein Gel in MES SDS running buffer and electroblotted onto Nitrocellulose membrane using iBlot2 gel transfer device (Life Technologies). All steps were performed according to the manufacturer's instructions. The resulting protein gels were then stained with the Coomassie Blue kit from Abcam, to determine differences in protein extracts between PBS and infected flies. We also used these gels to carry out western blot probing for protein with the HRP epitope, using the goat anti-HRP (123-165-021, Jackson ImmunoResearch Labs) at 1:100 and donkey anti-goat Alexa 568 antibody (Invitrogen) at 1:200. The bands in the protein gels that were positive for HRP and contained proteins with differences in quantities when compared with control and *FucTA* mutants were cut out and subjected to de-staining, reduction, alkylation, washing, trypsin digestion and peptide extraction followed by mass spectrometry (LC-MS/MS).

### Melanization assays

Adult haemolymph was collected as follows. Fifteen individuals were placed on a 10 µM filter of an empty mobicol spin column (MOBITEC), covered with glass beads and centrifuged for 20 min at 4°C at 2169 ***g***. Haemolymph was recovered in 50 µl protease inhibitor solution (Roche; one tablet dissolved in 4 ml PBS) and protein concentrations adjusted after a Bradford test. Sample volumes were adjusted to 200 µl in 5 mM CaCl_2_ solution (diluted in protease inhibitor solution, see above) and after addition of 800 µl of L-DOPA solution [20 mM in phosphate buffer (pH 6.6)] the samples were incubated at 29°C in the dark. After 30 min, the optical density at 492 nm was measured for each sample against a L-DOPA control containing no haemolymph. As activation of the proPO system was blocked by the presence of the protease inhibitor, the values reflect the *in vivo* PO activity at the time of infection. Melanization assays were repeated ten times.

## Supplementary Material

Supplementary information
