# Supplementary material for: A genetic screen in Drosophila reveals the role of fucosylation in host susceptibility to Candida infection
Source: Dis Model Mech. 2022 May 9;15(5):dmm049218. doi: 10.1242/dmm.049218 (PMC9118035; doi:10.1242/dmm.049218)
Supplement: Supplementary information [file dmm-15-049218-s1.pdf]

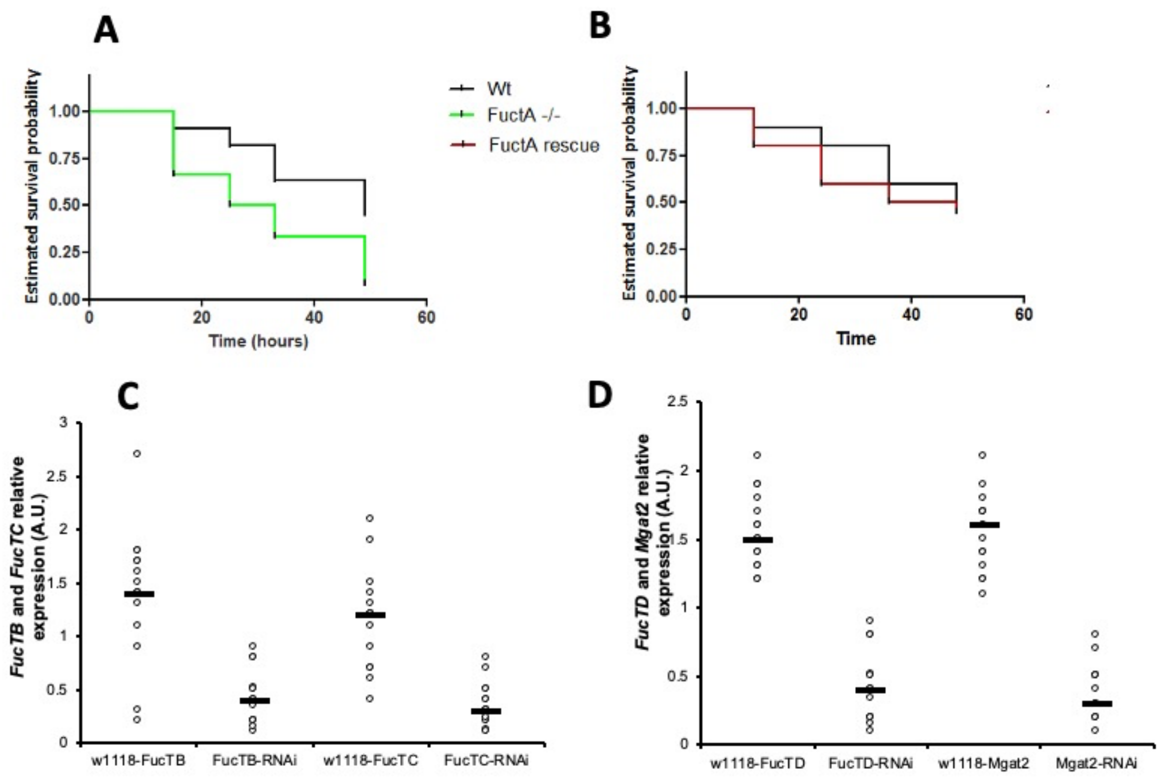

Fig. S1: FucTA is needed for flies to survive *C. albicans* infection. A. Flies homozygous for the FucTA mutant allele exhibited the same phenotype of susceptibility to *C. albicans* infection as the *c564-GAL4<UAS-FucTA*^{RNAi} flies when compared to the VDRC genetic background (used as "wild type" control). **B.** This was rescued when the transposable element in *FucTA*^{f03774} was precisely excised.

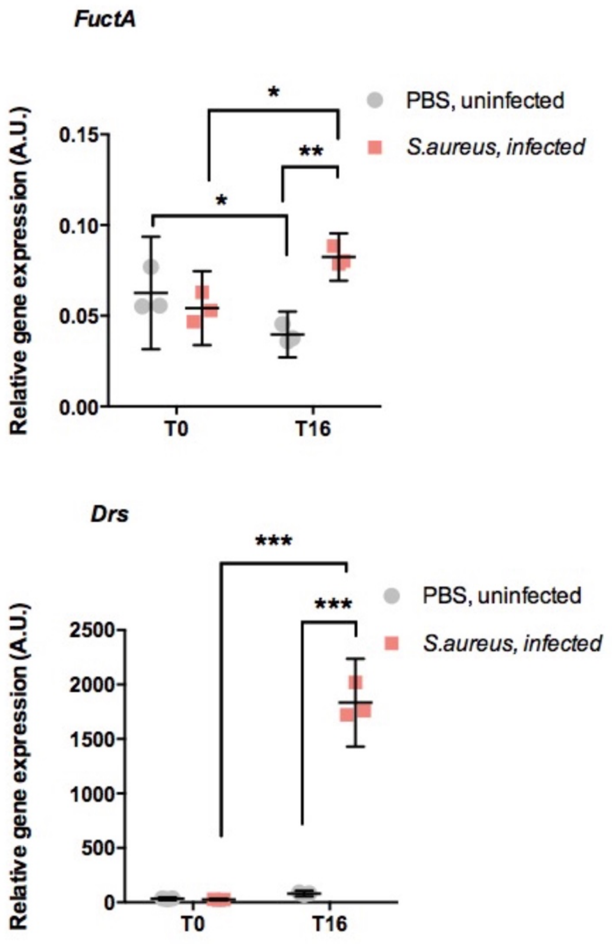

Fig. S2. *FucTA* transcription is induced by *S. aureus* infection. FucTA: Following *S. aureus* infection of *w*^{1118} flies, the levels of the *FucTA* transcript were significantly induced between T0 and T16 (hours) (*p<0.01), while at T16 *FucTA* gene expression was significantly higher than flies injected with sterile PBS (**p<0.001). *FucTA* transcription was significantly lower in T16 compared to T0 in PBS-injected flies (*p<0.01). To ascertain that infection was productive, we assayed expression of the AMP gene *drosomycin* (**Drs)**. *S. aureus* infection induced a robust activation of *drosomycin* expression compared to controls at T16 (***p<0.0001). Each dot is the average of 5 flies and the experiment was repeated three times (Student's t-test was used for comparisons).

Disease Models & Mechanisms • Supplementary information

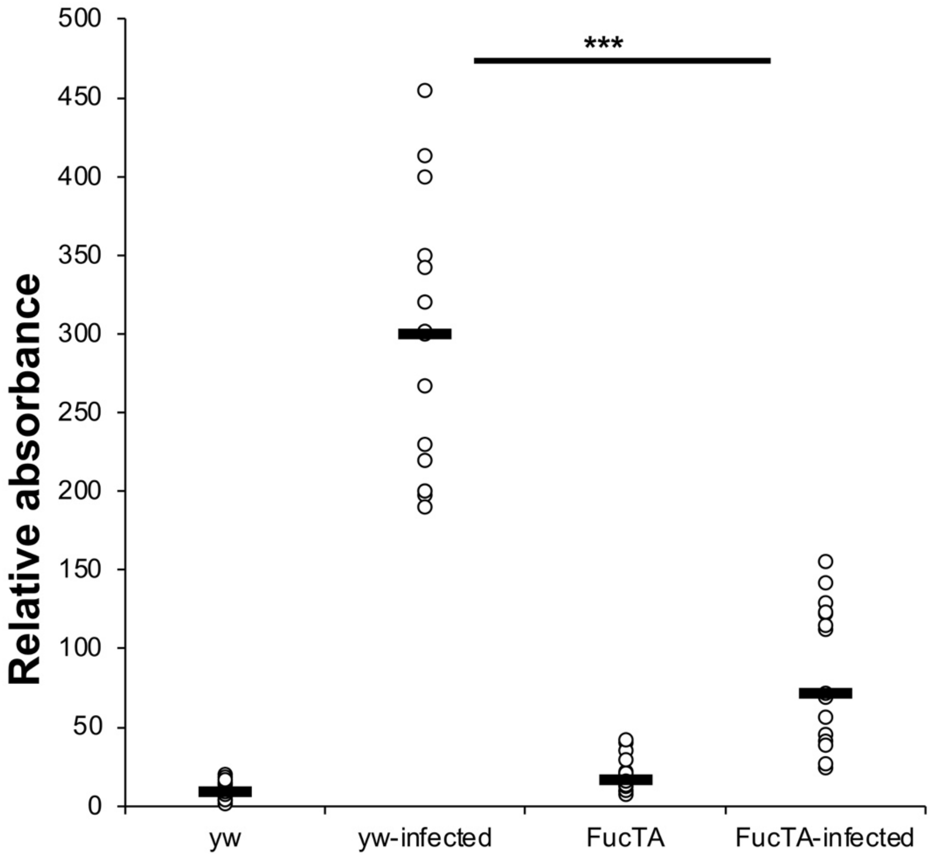

Fig. S3. *FucTA*^{f03774} infected flies have a lower level of hemolymph melanisation. Flies homozygous for the *FucTA*^{f03774} mutant allele exhibited a significantly lower level of melanisation in their hemolymph compared to *yw* controls (***p<0.0001), 30mins after they were infected with *S. aureus*. Each dot represents the average melanisation of 15 flies (n=15X10 independent experiments for each genotype/treatment). Student's t-test was used for comparisons.

Disease Models & Mechanisms • Supplementary information

Table S1. Infection survival data for RNAi screen (*C. albicans*)

Click here to download Table S1

Table S2. Infection survival data for RNAi screen (*S. aureus*)

Click here to download Table S2

Table S3. RNAi Screen Target Highlights

Click here to download Table S3

Table S4. Infection survival data for Fuc-TA-RNAi (CG6869).

Click here to download Table S4

Disease Models & Mechanisms • Supplementary information
